# Identification, Characterization and Functional Analysis of C-Class Genes Associated with Double Flower Trait in Carnation (*Dianthus caryphyllus* L.)

**DOI:** 10.3390/plants9010087

**Published:** 2020-01-10

**Authors:** Qijian Wang, Naizhen Dan, Xiaoni Zhang, Shengnan Lin, Manzhu Bao, Xiaopeng Fu

**Affiliations:** 1 Key Laboratory of Horticultural Plant Biology, College of Horticulture and Forestry Sciences, Huazhong Agricultural University, Wuhan 430070, China; qjwang@webmail.hzau.edu.cn (Q.W.); Yanxiuli@webmail.hzau.edu.cn (N.D.); zhangxiaoni@webmail.hzau.edu.cn (X.Z.); linshengnan@webmail.hzau.edu.cn (S.L.); mzbao@mail.hzau.edu.cn (M.B.); 2 Key Laboratory of Urban Agriculture in Central China (pilot run), Ministry of Agriculture, Wuhan 430070, China

**Keywords:** carnation, Caryophyllales, *AGAMOUS*, bioinformatics analysis, expression analysis, genetic transformation

## Abstract

Flowers with more petals are of more ornamental value. It is well known that *AGAMOUS* (*AG*) is the core member of the C-class gene which plays an essential role in double flower formation and identification of stamens and carpels in *Arabidopsis* *thaliana*. We searched C-class genes in the genome of the carnation, and found two *AG* orthologs (*DcaAGa*, *DcaAGb*). Phylogenetic analysis showed that the two genes were closely related to the *euAG* subclade. Then we searched the genomes of other Caryophyllales plants (*Beta vulgaris*, *Spinacia oleracea*, *Chenopodium quinoa*) for C-class genes, and found that their C-class genes all belonged to the *euAG* subclade. Semi-quantitative PCR (sq-PCR) analysis indicated that the expression of *DcaAG* genes in the single flower phenotype was higher than that in the double flower phenotype. Quantitative real-time RT-PCR (qRT-PCR) analysis showed that the expressions of *DcaAG* genes in the flower bud were significantly different from those in the root, stem, and leaf between the single and double flower phenotype carnations, and that *DcaAG* genes were specifically expressed in the stamen and carpel of carnation. Moreover, the expression of other floral organ identity genes (*AP1* and *AP2*, *PI* and *AP3*, *SEP1* and *SEP3* corresponding to the A-, B-, and E-class of genes, respectively) showed no significant difference in all floral organs between the single and double flower phenotype carnations, suggesting that C-class (*DcaAG*) genes might play an important role in the double flower phenotype in carnation. Petal loss or decrease, precocious flowering, silique shortening, and seed sterility were observed in *35S::DcaAGa* and *35S::DcaAGb* transgenic *Arabidopsis* plants. All these results show that *DcaAG* genes might affect the petal number negatively and have a specific function in stamen and carpel development in carnation.

## 1. Introduction

Flowers are important sexual organs of angiosperms, and the flower development process has been a research hotspot in ornamental plants for a long time. The molecular mechanism of the flower development in *Arabidopsis thaliana* [1] and *Antirrhinum majus* [2] is extremely clear, but little is known about the regulation network of flower development in many non-model plants. The ABC model explains the genetic regulatory mechanism of floral organs in *A. majus* and *Arabidopsis* [3]. Class A genes control the formation of the first whorl floral organ, class A and B genes work together to control petal formation, class B and C genes control stamen formation, and class C genes regulate the formation of carpel [4,5]. On the basis of the original ABC model, the ABCDE model is proposed with the additional class D genes function for ovule development and the class E genes function for all floral organs’ development [4,5,6].

The *AG* subfamily experienced many duplication events, but two of the duplication events are representative during the evolutionary process [7]. In the early evolution stage of core dicotyledonous plants, *AG* and *PLENA* (*PLE*) were parallel branches derived from a common ancestor after they experienced the gene duplication event. This duplication event resulted in the formation of the *euAG* subseries (*FARINELLI* (*FAR*) and *AG*) and the *PLE* subseries (*SHATTERPROOF1/2* (*SHP1/2*) and *PLE*) [7,8,9,10]. Another earlier duplication event occurred before the emergence of angiosperms, which resulted in the emergence of the C-class gene (*AG*) and D-class gene (*AGL11*) [7,10,11]. The mutations of *AG* and *PLE* led to consistent phenotypic features, the stamens converted into petals, together with the replacement of carpels by sepals [12,13]. In *A. majus*, although the *PLE* lineage gene (*PLE*) and *euAG* lineage gene (*FAR*) exhibited the partial redundant function of C-class genes [12,14], the function of *PLE* and *FAR* are slightly different. *PLE* functions to identify stamen and carpel, and to terminate floral meristem, while *FAR* plays an important role in male fertility [14]. In *Cyclamen persicum*, the repressive expression of *CpAG1* resulted in the conversion of stamens into petals, but the inhibited expression of *CpAG2* only caused incomplete formation of stamens and carpels. Although *CpAG1* and *CpAG2* had similar protein sequence, but they had different roles in whorl 3 and whorl 4 [15]. In *Phalaenopsis aphrodita*, two *AG* genes *PhalAG1* and *PhalAG2* belong to the C- and D-lineages, respectively. The expressions of *PhalAG1* and *PhalAG2* were much higher in flower buds than that in vegetative organs, and they played a redundant role in flower development [16]. In *Arabidopsis*, *AG* belonged to the C-class genes of the MADS-box family, played an essential role in the formation of floral meristem and sexual organs during flower development [17]. Previous studies reported that in C-class gene mutants of *Arabidopsis*, stamens are homologously transformed into petals, and *AG* inhibited A-class genes’ activity in petals and carpels, and that *AG* was able to identify the third and fourth whorls [18,19,20]. Moreover, some floral organ identity genes also play an important role in flower development. *APETALA1* (*AP1*) and *APETALA2* (*AP2*) are A-class genes in *Arabidopsis*, *AP1* functions to identify sepals and petals and *ap1* mutant phenotype shows the formation of leaf-like sepals [21]. With the exception of *AP2*, all A-, B-, C-, D-, and E- class genes are MADS-box genes. *AP2* is necessary for the specification of the first and second whorls [17,22]. *APETALA3* (*AP3*) and *PISTILLATA* (*PI*) represent B class genes, which play a major role in specifying petal and stamen identities in *Arabidopsis* [23,24]. *SEPALLATA1/2/3* (*SEP1/2/3*) are E-class genes, play a major role in the specification of all floral organs and floral determinacy [6,25,26]. Plants with a triple mutant of *sep1/2/3* show the phenomenon of all floral organs changing to sepals [26].

As one of the famous cut flowers, the carnation is widely used in daily life throughout the world. Recently, double flower trait have attracted researcher’s attention due to its improvement for ornamental value of many species such as carnation, rose, and lily. Many studies have been performed to clarify the molecular mechanism associated with double flower formation, such as *Arabidopsis* [22], *Eschscholzia californica* [27], and *Prunus lannesiana* [28]. In carnation, *D85* locus controlled flower phenotype and two SSR markers (CES0212 and CES1982) tightly linked to this locus were identified [29]. Despite the preliminary localization of the double flower phenotype and the genomic information in the carnation, suitable candidate genes cannot be screened at present due to the lack of chromosomal information in the genome [30].

In our study, firstly, we obtained two C-class genes in carnation, and found that the two C-class genes (*DcaAGa* and *DcaAGb*) belong to the *euAG* subclade. In order to determine whether the C-class genes in Caryophyllales all belong to the *euAG* subclade, C-class genes in *B. vulgaris*, *S. oleracea*, *C. quinoa* were searched and analyzed, and they all belong to the *euAG* subclade consistent with previous studies [31,32]. Subsequently, two C-class genes, *DcaAGa* and *DcaAGb* from two flower phenotype carnations were cloned and analyzed. The expressions of two *DcaAG* genes were analyzed using qRT-PCR and semi-quantitative PCR between single and double flower phenotype carnations, and the functions of these two genes were analyzed through overexpression of *DcaAGa* and *DcaAGb* in *Arabidopsis*. The expression analysis of other floral organ identity genes was performed in floral organs between single and double flower phenotype carnations by using qRT-PCR. Our data suggests that the two *DcaAG* genes might play a major role in affecting petal number, and that they play a specific role in regulating stamen and carpel development.

## 2. Results

### 2.1. Isolation and Sequence Analysis of the DcaAG Genes from Single and Double Flower Phenotype Carnations

**Figure 1:**
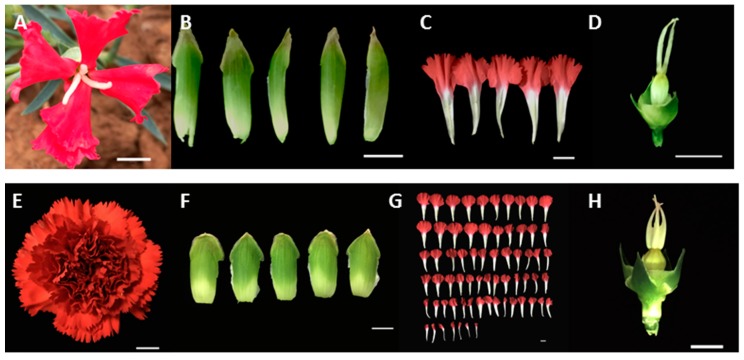
Characteristics of single (‘Da hong’) and double (‘Master’) flower phenotype carnations. (**A**–**D**) represent flower, sepals, petals, and carpel of ‘Da hong’, respectively. (**E**–**H**) represent flower, sepals, petals, and carpel of ‘Master’, respectively. ‘Da hong’ and ‘Master’ with petals of 5 and more than 40, respectively. Bar = 1 cm.

**Figure 2:**
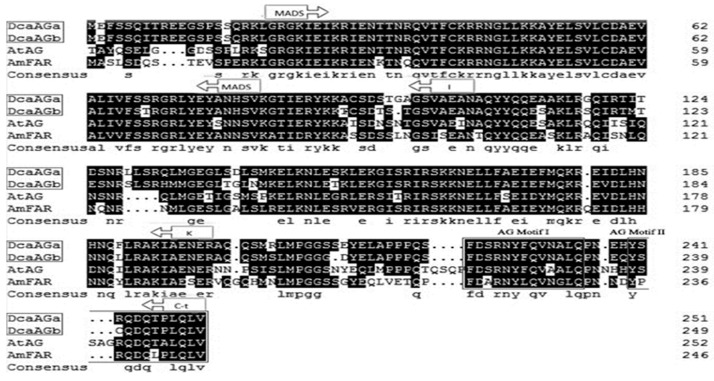
Protein sequence comparison of DcaAGa, DcaAGb, and the AG-related MADS domain proteins in *Arabidopsis thaliana* and *Antirrhinum majus*. The MADS domain, K domain, and I domain are marked with an arrow box. The AG motifs I and II in the C terminal region are boxed, highly conserved for AG-like protein.

Single flower phenotype carnation (‘Da hong’) which was collected in south China in 2015, has five petals (Figure 1, Table 1). Double flower phenotype carnation (‘Master’), a common cultivar, is wildly used in the cut flower market, has more than 40 petals (Figure 1, Table 1). Two *DcaAG* genes were identified from the carnation genome [30] and cloned from these two flower phenotype carnations, named *DcaAGa* and *DcaAGb*, respectively. The CDS sequences of *DcaAGa* and *DcaAGb* between two flower phenotype carnations were the same, respectively. The *DcaAGa* encoded a 251 amino acid protein, while the *DcaAGb* encoded a 249 amino acid protein (Figure 2), both of which contained the MADS domain (20–81), a short I region (82–105), a not highly conserved K domain (106–199), and the C-terminal region (200–251) with AG motifs I and II (Figure 2).

Bioinformatics analysis revealed that the molecular weights (MW) of DcaAGa and DcaAGb were 28.73 and 28.49 kDa, respectively (Table 2), and the isoelectric points (pI) of DcaAGa and DcaAGb were 9.43 and 9.28, respectively (Table 2). Prediction of subcellular localization revealed that both DcaAGs were predicted to be located in the nucleus (Table 2).

**Figure 3:**
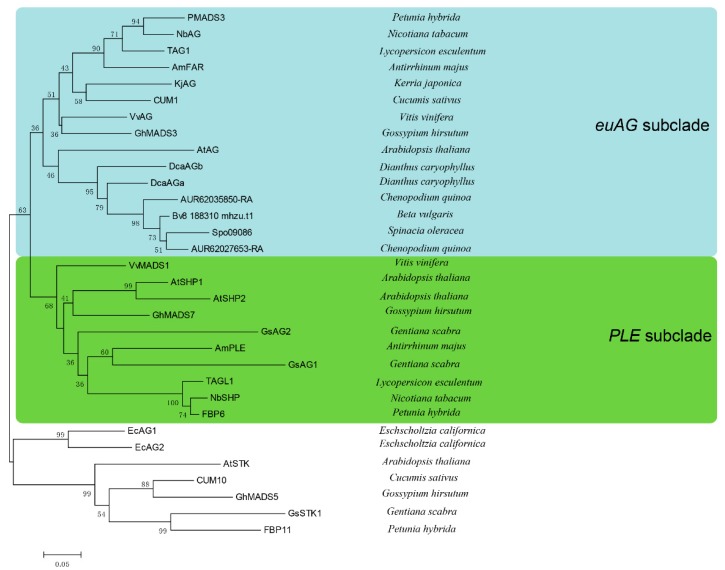
Phylogenetic tree showing the relationship between *DcaAG* genes and major C/D-class MADS-box proteins of other plants. *DcaAG* genes and C-class genes in Caryophyllales plants (*Beta vulgaris*, *Spinacia oleracea*, and *Chenopodium quinoa*) divide into *euAG* subclade rather than *PLE* subclade. MEGA 5.0 is used to construct phylogenetic tree, the maximum evolution method is used to infer evolutionary relationships. The phylogenetic tree is estimated by setting 2000 bootstrap replicates. The numbers below the branches refer to bootstrap value.

Phylogenetic analysis showed that *DcaAGa* and *DcaAGb* all fall into the *euAG* branch (Figure 3). Interestingly, our result was consistent with the previous study findings that *AG* homologs from Caryophyllales belonged to only the *euAG* subclade rather than the *PLE* subclade [7,31,32]. We identified one, one, and two C-class genes from *B. vulgaris*, *S. oleracea* and *C. quinoa*, respectively, and found that they all belonged to the *euAG* subclade (Figure 3).

### 2.2. Expression Analysis of DcaAG Genes in Different Tissues and Floral Organs of Carnations

**Figure 4:**
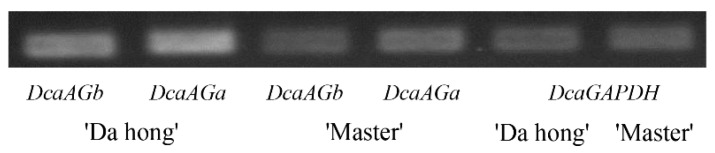
Semi-quantitative PCR (sq-PCR) analysis of two *DcaAG* genes in single and double flower phenotype carnations. The expression of both *DcaAG* genes in the double flower phenotype carnation were lower than that in the single flower phenotype carnation.

Semi-quantitative PCR analysis indicated that the expression levels of *DcaAG* genes were lower in the double flower phenotype carnation than that in the single flower phenotype carnation, and that the expression patterns of *DcaAGa* and *DcaAGb* in these two flower phenotype carnations were basically consistent, while the expression of the former was slightly higher than that of the latter (Figure 4).

**Figure 5:**
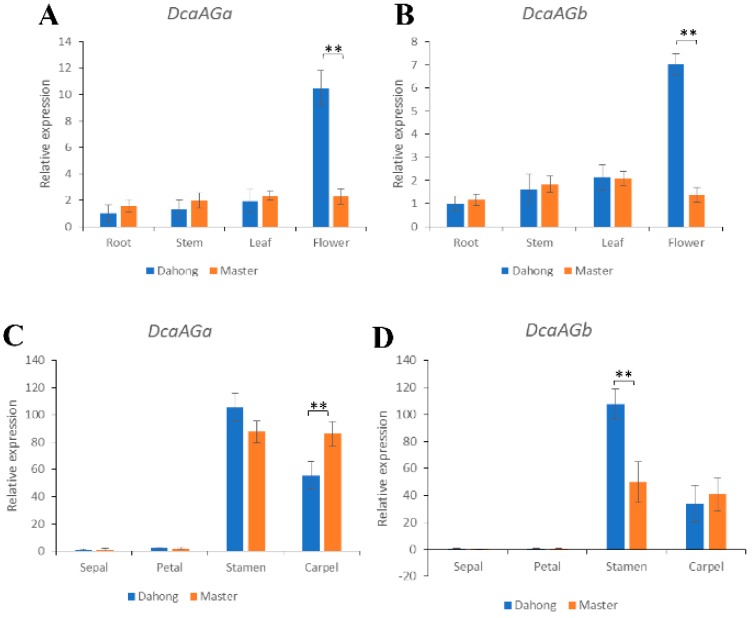
Quantitative PCR analysis of two *DcaAG* genes at different tissues (**A**,**B**) and floral organs (**C**,**D**) in single and double flower phenotype carnations. In different tissues, the expression of *DcaAGa* (**A**) and *DcaAGb* (**B**) were obviously different in the flower bud, and no significant difference was detected in the root, stem and leaf between two flower phenotype carnations. In floral organs, the expression of *DcaAGa* (**C**) and *DcaAGb* (**D**) were exclusively expressed in the stamen and carpel, and low expression levels were detected in the sepal and petal. *DcaAGa* (**C**) and *DcaAGb* (**D**) showed significant differential expression in the carpel and stamen between two flower phenotype carnations, respectively. ** indicates *p* < 0.01 by student’s *t*-test.

To examine the expression of *DcaAG* genes between the single and double flower phenotype in different tissues, qRT-PCR analysis was performed. Significant differential expression was detected in the flower bud (Figure 5). The expression levels of *DcaAG* genes in the root and stem of the single flower phenotype carnation were slightly lower than those of the double flower phenotype carnation (Figure 5). On the contrary, the expression levels of *DcaAG* genes in the flower bud of the single flower phenotype carnation plant were much higher than that of the double flower phenotype carnation (Figure 5), which indicated that *DcaAG* genes might play a major role in the formation of differential characteristics between single and double flower phenotype carnations.

The expression patterns of *DcaAG* genes between the two flower phenotypes in different floral organs (carpel, stamen, petal, and sepal) were analyzed by qRT-PCR. *DcaAG* genes were exclusively expressed in the carpel and stamen, while extremely low expression levels were detected in the sepal and petal (Figure 5). The expression levels of *DcaAG* genes were higher in the stamen of the single flower phenotype carnation than that of the double flower phenotype carnation (Figure 5). On the contrary, the expression levels of *DcaAG* genes were lower in the carpel of the single flower phenotype carnation than that of the double flower phenotype carnation (Figure 5).

### 2.3. Expression Pattern Analysis of A-, B-, and E-Class Genes in Single and Double Flower Phenotype Carnations

**Figure 6:**
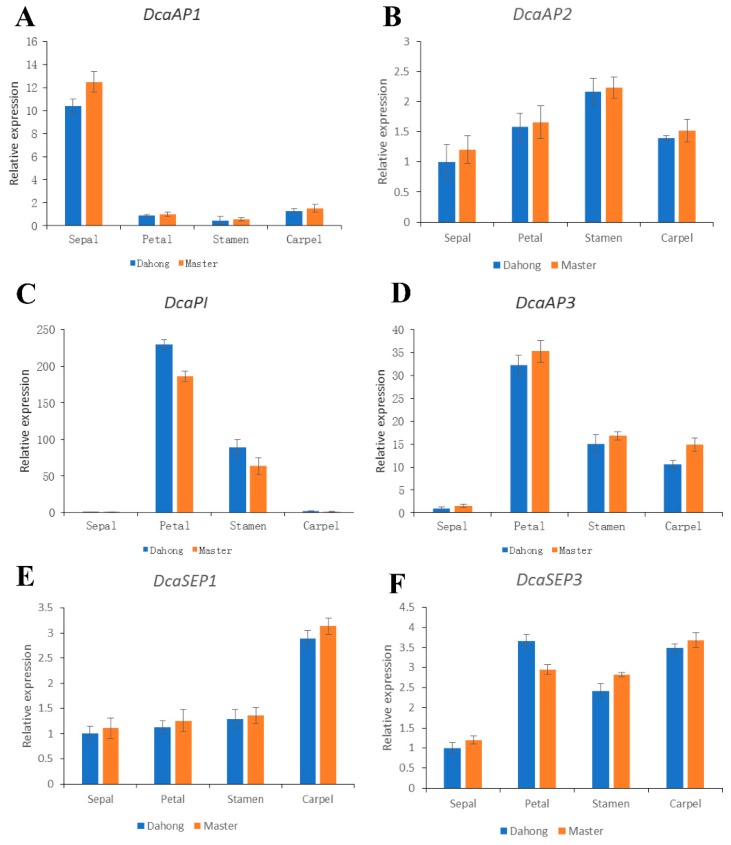
Quantitative PCR analysis of floral organ identity genes at different floral organs in single and double flower phenotype carnations. *DcaAP1* (**A**) had higher expression in the sepal than that in petal, stamen, and carpel. *DcaAP2* (**B**) expressed in all floral organs stably. *DcaPI* (**C**) and *DcaAP3* (**D**) had higher expression in the second and third whorls than that in the first and fourth whorls. *DcaSEP1* (**E**) and *DcaSEP3* (**F**) expressed relatively smoothly in all floral organs between single and double flower phenotype carnations. The expression of *DcaAP1* (**A**), *DcaAP2* (**B**), *DcaPI* (**C**), *DcaAP3* (**D**), *DcaSEP1* (**E**), and *DcaSEP3* (**F**) had no significant difference in sepal, petal, stamen and carpel between two flower phenotype carnations. Student’s *t*-test was used to determine significant differences in expression.

In order to investigate the expression levels of the floral organ identity genes between single and double flower phenotype carnations quantitative RT-RCR was performed to analyze the expression pattern of *DcaAP1*, *DcaAP2*, *DcaAP3*, *DcaPI*, *DcaSEP1*, and *DcaSEP3*. *DcaAP1* had especially higher expression in the sepal than that in the petal, stamen, and carpel, and expressed slightly lower in all floral organs of the single flower phenotype carnation compared to that of the double flower phenotype carnation (Figure 6A). *DcaAP2* expressed in all floral organs stably and was slightly lower in all floral organs of the single flower phenotype carnation than that of the double flower phenotype carnation (Figure 6B). *DcaPI* and *DcaAP3* both had higher expression in the second and third whorls than that in the first and fourth whorls, and there was no significant difference in expression between single and double flower phenotype carnations (Figure 6C,D). The expression pattern of *DcaSEP1* and *DcaSEP3* were relatively smooth in all floral organs between single and double flower phenotype carnations (Figure 6E,F). The huge difference of expression in C-class genes and no significant difference of expression in A-, B-, and E-class genes between single and double flower phenotype carnations indicated that the C-class genes may result in the differential characteristics between single and double flower phenotype carnations. A previous study on *Kerria japonica* revealed that repressing the expression of *Df-KjAG* could affect the expression of other floral organ identity genes, such as *KjAGL2*, *KjAGL9*, *KjAP1*, *KjAP2*, *KjAP3*, and *KjPI* in the double flower *K.japonica* [33], which is different from the situation in carnation.

### 2.4. Phenotypes of DcaAG Genes Overexpression in *Arabidopsis*

**Figure 7:**
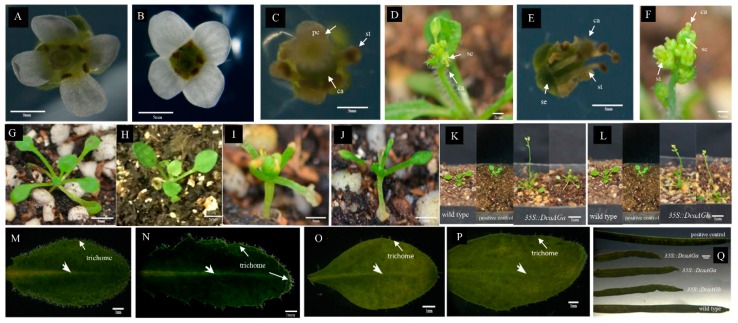
Phenotypic analysis of transgenic plants by overexpressing *DcaAG* genes in *Arabidopsis*. The flower of the wild-type *Arabidopsis* (A) and positive control group (**B**) (*Arabidopsis* transformed with empty vector). The petals of *35::DcaAGa* (**C**,**D**) and *35::DcaAGb* (**E**,**F**) transgenic plants is decreased or almost disappears compared to the wild type plant and positive control group. Phenotype of leaves in the early developmental stage of wild type plants (**G**), positive control group (**H**), *35::DcaAGa* (**I**) and *35::DcaAGb* (**J**) transgenic plants, the transgenic plants display abnormal growth of narrow and curly leaves. *35::DcaAGa* (**K**) and *35::DcaAGb* (**L**) transgenic plants flowering earlier than that of the wild type plant and positive control group. Trichome on the leaf surface margin and vein in *35::DcaAGa* (**O**) and *35::DcaAGb* (**P**) transgenic plants are decreased compared to wild type plant (**M**) and positive control group (**N**). The siliques are shorter, wrinkled with convex near the top in transgenic plants, compared to wild type plants (**Q**). se: sepal, pe: petal, st: stamen, ca: carpel. Bar = 5 mm in A–J, and Q. Bar = 1 cm in K and L. Bar = 1 mm in M–P.

We investigated the function of *DcaAG* genes in flower initiation and development by overexpressing *DcaAG* genes in *Arabidopsis*. Twenty independent *35S::DcaAGa* and 33 independent *35S::DcaAGb* transgenic *Arabidopsis* plants were obtained. The expression levels of *DcaAGa* and *DcaAGb* in the transgenic *Arabidopsis* lines were tested by semi-quantitative PCR. Both *DcaAG* genes were found to express in transgenic plants (Figure S1). The 15 independent *35S::DcaAGa* and 14 independent *35S::DcaAGb* transgenic plants showed severe phenotypic alteration (Figure 7), namely, their petals became significantly shorter and even almost disappeared, and a large number of deformed carpels were observed (Figure 7C–F). All the transgenic plants displayed abnormal growth of narrow and curly leaves in the early developmental stage (Figure 7I–J). These transgenic plants flowered earlier than the wild type plants and the positive control group (Figure 7K,L). All the transgenic plants showed a significant decrease in trichome on the leaf surface margin and vein (Figure 7O–P). The seeds of *35S::DcaAGa* and *35S::DcaAGb* transgenic lines were sterile or with low activity, and their siliques were shorter, wrinkled, and convex near the top (Figure 7Q). These results indicated that overexpression of *DcaAG* genes in *Arabidopsis* lead to a series of effects on *Arabidopsis* development, especially on flower development.

### 2.5. Expression Analysis in Transgenic Arabidopsis

**Figure 8:**
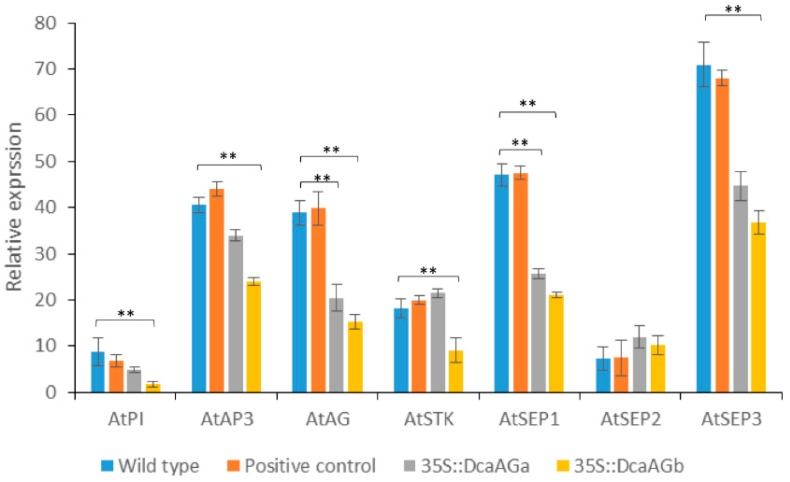
Quantitative PCR analysis of *AtPI*, *AtAP3*, *AtAG*, *AtSTK*, *AtSEP1*, *AtSEP2*, and *AtSEP3* in wild type, positive control group, *35::DcaAGa* and *35::DcaAGb* transgenic *Arabidopsis*. The expression of *AtPI*, *AtAP3*, *AtAG*, *AtSTK*, *AtSEP1*, and *AtSEP3* in transgenic *Arabidopsis* show a significant difference, compared to the wild type and positive control group plant. ** indicates *p* < 0.01 by student’s *t*-test. Positive control represents the plants are transformed with pCAMBIA2300s vector.

The expression levels of floral organ identity genes in transgenic *Arabidopsis* were detected by qRT-PCR analysis. The results showed that the expressions of *AtPI*, *AtAP3*, *AtAG*, *AtSEP1,* and *AtSEP3* were down-regulated in the *35S::DcaAGa* and *35S::DcaAGb* transgenic T1 lines, while the expression of *AtSEP2* was up-regulated, compared with the wild type plants and positive control group (Figure 8). The expression of *AtSTK* was slightly higher in the *35S::DcaAGa* transgenic plant than that in the wild type plants and positive control group, and was down-regulated in *35S::DcaAGb* transgenic plant (Figure 8). It indicated that the overexpression of *DcaAG* genes had an effect on genes related to flower development in *Arabidopsis*.

## 3. Discussion

C-class genes have been identified from the genome of the carnation and cloned from single and double flower phenotype carnations. No difference was observed in the CDS sequence of *DcaAG* genes between the two flower phenotype carnations. The amino acid sequence consistency of *DcaAGa* and *DcaAGb* achieves 89%, with a consistency of 98% at the N terminal and M domain, 79% in the I domain, 82% in the K domain, and 88% in the C terminal. Moreover, the CDS sequences of *DcaAGa* and *DcaAGb* exhibit a similarity as high as 71% to that of *AG* in *Arabidopsis*, while 69% and 66% similarity to that of *FAR* and *PLE* in *A. majus*, respectively. This suggests that the domains of *DcaAG* genes are relatively conservative during plant evolution.

In *Arabidopsis*, *AG* is one of the key genes for double flower formation. *SHP* was not physically present in the meristem or primordia cells at the investigated time, indicating that *AG* is the only C function factor in the process of floral primordia development [34,35]. The change in expression pattern is reported to lead to subfunctionalization of C function genes [36,37]. Semi-quantitative PCR analysis shows that the expression levels of *DcaAG* genes are lower in the double flower phenotype carnation than that of the single flower phenotype carnation (Figure 4), suggesting *DcaAG* genes in the carnation might be a negative regulator of double flower formation, which has been proved in many other species, such as *Arabidopsis* [38], Rose [39], and *Tricyrtis macranthopsis* [40]. The expression pattern of *DcaAG* genes in different tissues reveals a larger difference in the flower bud than that in the root, stem and leaf between two flower phenotype carnations (Figure 5), which indicates that the inhibition of the *DcaAG* gene might have an important effect on the flower development of the double flower phenotype carnation. This situation in the carnation is also reported in the studies of Rose [41], Ranunculids [18], and Cyclamen [15]. The expression pattern of *DcaAG* genes in different floral organs of two flower phenotype carnations indicates that the *DcaAG* genes are both expressed in the third and fourth whorls, but nearly not expressed in the first and second whorls (Figure 5). Conjointly with the expression analysis of *DcaAG* genes and the floral organ identity genes in floral organs between two flower phenotype carnations, the decreased expression of *DcaAG* genes in the double flower phenotype carnation corresponds to no significant difference in expression of other floral organ identity genes between the two flower phenotype carnations (Figure 5, Figure 6 ), indicating that C-class (*DcaAG*) genes might have an important role in double flower formation in the carnation. Moreover, the expression of *DcaAGa* is slightly higher than that of *DcaAGb* (Figure 4) and both *DcaAG* genes have a similar expression pattern in the two flower phenotype carnations (Figure 4 and Figure 5), which is similar to the expression pattern of *EScaAG1*, *EScaAG2* in *E. californica* [27] and *MtAGa*, *MtAGb* in *Medicago truncatula* [42], suggesting that two *DcaAG* genes might have a functional redundancy in regulating the flower organs in plants. However, the different expression level between two *DcaAG* genes might be due to their different regulation roles in exerting C function.

Compared to wild type *Arabidopsis*, early flowering is observed as a novel phenomenon in *35S::DcaAGa* and *35S::DcaAGb* transgenic *Arabidopsis* plants which is rarely reported in other species (Figure 7K,L). The phenotypes of *35S::DcaAGa* and *35S::DcaAGb* transgenic *Arabidopsis* plants exhibit petal loss, short silique, and almost seed sterility (Figure 7C–F,Q). These results indicate that overexpression of *DcaAG* genes in *Arabidopsis* cause the abnormal development in the third and fourth whorls and inhibit growth of petals in transgenic plants. The phenotypes of *35S::DcaAGa* and *35S::DcaAGb* transgenic plants are highly similar (Figure 7), which might be attributed to the similar C-class function of two *DcaAG* genes. *AG* and *FAR* are orthologue genes [7,43]. The flower of the *FAR* mutant plant shows stamen sterility in *A. majus*, indicating that *FAR* has an important role in promoting stamen development [44], while *AG* plays an essential role in stamen and carpel development [23]. Our results suggest that both *DcaAGa* and *DcaAGb* in carnation could regulate the stamen and carpel in a different manner to the *euAG* genes (*AG* and *FAR*) [7].

In this research, phylogenetic analysis indicates that both *DcaAGa* and *DcaAGb* fall into the *euAG* subclade (Figure 3). C-class genes in other Caryophyllales plants fall into the same clade as *DcaAG* genes by phylogenetic analysis (Figure 3). Our findings are in agreement with previous study shows that *AG/FAR* and *SHP*/*PLE* represent two distinct paralogous lineages rather than single genetic orthologs [7,31,32]. The *AG/FAR* subgroup functions in identifying male and female organs, while the *SHP/PLE* subgroup plays an important role in the identification of floral organs in *A. majus* and *Arabidopsis* [12,17]. According to the phylogenetic analysis, expression analysis of *DcaAG* genes (Figure 3, Figure 4 and Figure 5) and the phenotype of the *35S::DcaAGa* and *35S::DcaAGb* transgenic *Arabidopsis* plants (Figure 7), we speculate that members of the *SHP/PLE* may disappear in some species of Caryophyllales, and only genes in the *AG/FAR* subgroup perform C-class function. *AG* and *SHP1/2* are the core C-class MADS-box genes in *Arabidopsis*, which is unlike many species with two C-class MADS-box genes, such as *A. majus* (*PLE/FAR*) [14], tomato (*TAG1/TAGL1*) [45], and cyclamen (*CpAG1/CpAG2*) [15]. In cyclamen, *CpAG1* and *CpAG2* have their own distinct roles in the stamen and carpel, respectively [15]. The situation in carnation is slightly different to cyclamen, the functions in the stamen and carpel of *DcaAGa* and *DcaAGb* are similar (Figure 7) which may indicate that *DcaAGa* and *DcaAGb* performed C function redundantly in carnation, and *DcaAGa* exhibits a stronger function than *DcaAGb* according to the expression pattern of the two *DcaAG* genes (Figure 4, Figure 5). Overall, our results indicate that C-class genes in the carnation all belong to the *euAG* subclade, and that *DcaAG* genes in the carnation play an essential role in regulating the formation of the stamen and carpel, especially in regulating the stamen, silique, and in seed development. Also, these two genes may have an important role in regulating petal number.

## 4. Materials and Methods

### 4.1. Plant Materials and RNA Sample Preparation

Two varieties of carnations, ‘Master’ (double flower phenotype) and ‘Da Hong’ (single flower phenotype), with different flower phenotype and biological characteristics (Table 1, Figure 1) were planted in Huazhong Agricultural University, China. Young root, stem, leaf, flower bud, and floral organs (sepal, petal, stamen and carpel) of two flower phenotype carnations were collected and frozen immediately in liquid nitrogen, and then stored at −80 °C for RNA extraction. RNA was extracted in the method described in a previous study [46]. *A. thaliana* Columbia (Col) ecotype plants were grown under a long-day regime (22 °C, 16/8 h light/dark) with light intensity of 8000 lx and humidity of 75%.

### 4.2. Search for C-Class Genes in the Genome of Caryophyllales Plants and Molecular Cloning of DcaAG Genes in Single and Double Flower Phenotype Carnations

The protein sequence of AG and SHP1/2 from *Arabidopsis*, downloaded from the TAIR website (http://www.arabidopsis.org/), were used to search C-class genes in the genome of carnation (http://carnation.kazusa.or.jp/), *S. oleracea* (http://www.spinachbase.org), *C. quinoa* (https://www.cbrc.kaust.edu.sa/chenopodiumdb/) and *B. vulgaris* (http://bvseq.boku.ac.at/index.shtml) by blastp (e-value 1^e−6^) program. The protein sequences obtained from these databases were aligned by DNAMAN 6.0 software. Those protein sequences without AG conserved domain were filtered. The flower bud’s cDNA of two flower phenotype carnations was used as a template for amplification of the target fragment. The CDS sequences of *DcaAGa* and *DcaAGb* in the carnation’s genome and Primer Premier 5.0 were used to design cloning primers. Primers are listed in Supplementary Table S1. PCR program was performed as follows: 4 min at 94 °C for 1 cycle, followed by 35 cycles at 94 °C for 30 s, 55 °C (*DcaAGa*)/58 °C (*DcaAGb*) for 30 s, and 72 °C for 1 min, and followed by 10 min at 72 °C for 1 cycle. A fragment of PCR product was collected and cloned into the pMD18-T vector (Takara, Japan, http://www.takara.com.cn), and then connected to Sangon Biotech (Shanghai) for sequencing.

### 4.3. Bioinformatics and Phylogenetic Analysis

The putative protein sequences were obtained from translation of CDS sequence of *DcaAGa* and *DcaAGb*. ProtParam (http://web.expasy.org/protparam/) was used to predicate molecular weight (MW) and isoelectric point (pI) of the amino acid sequences with default parameters. The predication of subcellular localization was analyzed by Plant-mPloc [47].

C-class genes in Caryophyllales plants (carnation, *B. vulgaris*, *S. oleracea* and *C. quinoa*) and the other major C/D-class MADS-box proteins in *Arabidopsis*, *A. majus*, *Lycopersicon esculentum*, *Petunia hybrid*, *Nicotiana tabacum*, *Eschscholtzia californica*, *Vitis vinifera*, *Kerria japonica*, *Cucumis sativus*, *Gossypium hirsutum*, and *Gentiana scabra* were used for phylogenetic analysis by using MEGA 5.0 [48]. Full-length protein sequences were aligned by MUSCLE with default parameters. The maximum evolution method with bootstrap of 2000 replicates and the Nearest-Neighbor-Interchange (NNI) algorithm were used to evaluate evolutionary relationships.

The accession number of the proteins used in the phylogenetic analysis are as follows: EcAG1 (DQ088996); EcAG2 (DQ088997); AtAG (X53579); AmFAR (AJ239057); AtSTK (NP_001329612); TAG1 (L26295); PMADS3 (X72912); NbAG (JQ699177); AtSHP1 (M55550); AtSHP2 (M55553); AmPLE (S53900); TAGL1 (AY098735); FBP6 (X68675); FBP11 (CAA57445); NbSHP (JQ699178); VvAG (NM_001281168); VvMADS1 (NP_001268105); KjAG (AMQ23646); CUM1 (AAC08528); CUM10 (AAC08529); GhMADS3 (XP_016711294); GhMADS5 (ABM69043); GhMADS7 (ABM69045); GsAG1 (LC022775); GsAG2 (LC022779); GsSTK1 (LC022768). All the protein sequences were downloaded from the Phytozome website (https://phytozome.jgi.doe.gov/pz/portal.html) and listed in Supplementary Table S2.

### 4.4. Quantitative PCR and Semi-Quantitative PCR Analysis of DcaAG Gene Expression in Single and Double Flower Phenotype Carnations

The RNA extracted from different tissues (root, stem, leaf, and flower bud), and floral organs (sepal, petal, stamen, and carpel) were used for qRT-PCR analysis to investigate the expression pattern of *DcaAGa* and *DcaAGb* in two flower phenotype carnations. Then, the expression patterns of A- (*AP1*, *AP2*), B- (*PI*, *AP3*), and E-class (*SEP1*, *SEP3*) genes in floral organs (sepal, petal, stamen, and carpel) were analyzed by qRT-PCR. A-, B-, and E-class Genes were identified in a previous study [46]. Quantitative real-time PCR analysis was performed as described previously [46]. The experiment with each sample was conducted with three biological replicates. The *DcaGAPDH* (glyceraldehyde-3-phosphate dehydrogenase) and *β*-*actin* were used as housekeeping genes. The comparative CT(2^−ΔΔCT^) method was used to calculate the relative expression value.

For semi-quantitative PCR analysis, the cycle parameters were 95 °C/4 min for 1 cycle, 95 °C/30 s, 57 °C/30 s, 72 °C/20 s for 26 cycles, and followed by 10 min at 72 °C for 1 cycle. *DcaGAPDH* was used as reference gene. Primer Premier 5.0 was used to design qRT-PCR and semi-quantitative PCR primers. The sequences of primers are listed in Supplementary Table S3.

### 4.5. Overexpression of DcaAGa and DcaAGb in *Arabidopsis*

*BamH I* and *SalI* were used to digest pMD18-T vector containing the full-length CDS sequence of two *DcaAG* genes. The full-length CDS sequence of *DcaAG* genes were subcloned into binary vector pCAMBIA2300s under the control of cauliflower mosaic virus 35S promoter in sense orientation. The *35S::DcaAG* genes were introduced to the *Agrobacterium tumefaciens* strain GV3101, which were subsequently transformed into *Arabidopsis* plants by the floral dip procedure. Total RNA in the flower bud of *35S::DcaAGa* and *35S::DcaAGb* transgenic plants was extracted, and used for semi-quantitative PCR and qRT-PCR analysis. The expression levels of *DcaAGa* and *DcaAGb* in the transgenic *Arabidopsis* lines were tested by semi-quantitative PCR, and *AtACT2* was used as reference gene. The PCR program was performed as follows: 95 °C/3 min for 1 cycle, 95 °C/30 s, 58 °C/30 s, 72 °C/20 s for 27 cycles, and followed by 8 min at 72 °C for 1 cycle. The qRT-PCR analysis of genes associated with flower development including *PI*, *AP3*, *AG*, *SEEDSTICK* (*STK*), *AG*, *SEP1*, *SEP2*, and *SEP3* were performed to observe the expression difference between wild type, positive control and transgenic *Arabidopsis* plants. The *AtACT2* and *AtACT11* were used as housekeeping genes. Primer Premier 5.0 was used to design semi-quantitative PCR and qRT-PCR primers. Sequences of primers are listed in Supplementary Table S4.

## 5. Conclusions

In this research, we identified two C-class genes (*DcaAGa* and *DcaAGb*) in carnation and found that they fall intothe *euAG* subclade rather than the *PLE* subclade. *DcaAGa* and *DcaAGb* are cloned from single and double flower phenotype carnations and the CDS sequence of *DcaAGa* and *DcaAGb* show no difference between the two flower phenotype carnations, respectively. The expression of *DcaAG* genes in different tissues (root, stem, leaf, and flower bud) shows that *DcaAG* genes have a larger difference in flower bud than that in the root, stem and leaf between single and double flower phenotype carnations. The expression levels of *DcaAG* genes and other floral organ identity genes (A-class of *AP1* and *AP2*, B-class of *AP3* and *PI*, E-class of *SEP1* and *SEP3*) were analyzed in floral organs (sepal, petal, stamen, and carpel) between two flower phenotype carnations. This revealed that only *DcaAG* genes show significant differential expression in stamen and carpel. Overexpression of *DcaAGa* and *DcaAGb* in *Arabidopsis* show that *DcaAGa* and *DcaAGb* play an important role in stamen and carpel development, regulating the petal number negatively, while having a similar function which indicates a redundant function between them. This study provides a foundation for future study on the double flower trait in the carnation.

## Figures and Tables

**Table 1:** Information of the experimental materials.

Flower Phenotype	Name of Material	Petal Number	Color
Single	‘Da-Hong’	5	Red
Double	‘Master’	>40	Dark red

**Table 2:** Protein properties of DcaAGa and DcaAGb.

Gene	Accession ID	AA Length	Molecular Weight (kDa)	Isoelectric Point	Subcellular Localization	Signal Peptide
DcaAGa	Dca35398.1	251	28.72751	9.43	nucl	NO
DcaAGb	Dca50159.1	249	28.48724	9.28	nucl	NO

AA: Amino acid; Predication of subcellular localization was analyzed by Plant-mPloc.

## Supplementary Materials

The following are available online at https://www.mdpi.com/2223-7747/9/1/87/s1, Table S1: The sequences of primers used for cloning *DcaAG* genes, Table S2: Protein sequences used for phylogenetic analysis, Table S3: The sequences of primers used for semi-quantitative PCR and qRT-PCR analysis in carnation. Table S4: The sequences of primers used for semi-quantitative PCR and qRT-PCR in transgenic *Arabidopsis*. Figure S1: Semi-quantitative PCR analysis of two *DcaAG* genes in different transgenic *Arabidopsis* lines.
