# Supplementary material for: Identification, Characterization and Functional Analysis of C-Class Genes Associated with Double Flower Trait in Carnation (Dianthus caryphyllus L.)
Source: Plants (Basel). 2020 Jan 10;9(1):87. doi: 10.3390/plants9010087 (PMC7020439; doi:10.3390/plants9010087)
Supplement: Supplementary file 1 [file plants-09-00087-s001.pdf]

plants

MDPI

Supplementary Material

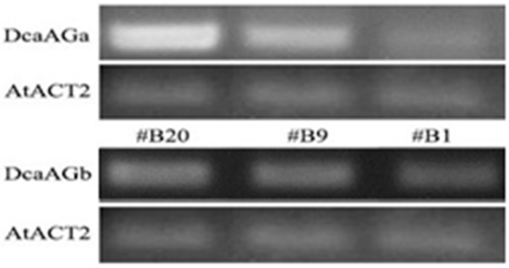

Figure S1. Semi-quantitative PCR analysis of two *DcaAG* genes in different transgenic *Arabidopsis* lines. Both *DcaAG* genes were found to express in transgenic plants. #B20, #B9, and # B1 represent the transgenic *Arabidopsis* plant with severe, moderate, and weak phenotypic variation, respectively. *AtACT2* was used as reference gene.

Table S1: The sequences of primers used for cloning *DcaAG* genes.

Gene name	Oligo Name	Primer Sequence（5′–3′）
DcaAGa	*DcaAGa*-F	ATGGAATTTTCAAGCCAAATAACTAGG
	DcaAGa-R	TCAAACAAGTTGAAGAGGTGTTTGG
DcaAGb	*DcaAGb*-F	ATGGAGTTTTCAAGCCAAATTAC
	DcaAGb-R	TTACACAAGTTGGAGAGGAGTTT

Table S2: Protein sequences used for phylogenetic analysis.

Gene	Accession Number	Species	Protein Sequence
EcAG1	DQ088996	*Eschscholtzia californica*	TDFQSQVTEDSSRRKMGRGKIEIKRIENTTNRQVTFCK RRNGLLKKAYELSVLCDAEVALIVFSTRGRLYEYANNS VKSTIERYKKTCADPSNSACASEANTQFYQQEATKLR QQIGILQNSNRNLMGEAISTMSVKELKQLENRLEKGIS RIRSKKNELLFAEIEYMQKREIDLQNDNMYLRAKIAD NERAQQQMSLMPGNEYEGMTSSGYDSRNFLQVNLLQ SSSQHYSHQEQTTLQLG
EcAG2	DQ088997	*Eschscholtzia californica*	TDFPNQEREISSGRKMGRGKIEIKKIENTTNRQVTFCKR RNGLLKKAYELSVLCDAEVALVVFSNRGRLYEYANNS VRSTIERYKKTCADPSNSSCSSEANIQFFQQEASKLRQQ IAILQNSNRHLMGESLSSMNVKELKQLETRLEKGISRIR SKKNELLFAEIELMQKREIDLQNHNMYLRSKIAEKERA EQHMRLTPGNEYNDMISRNFLQVNFLQSSNHQYSHQ EQTSLQLG
AmFAR	AJ239057	*Antirrhinum majus*	MASLSDQSTEVSPERKIGRGKIEIKRIENKTNQQVTFCK RRNGLLKKAYELSVLCDAEVALVVFSSRGRLYEYANN SVKATIDRYKKASSDSSLNGSISEANTQYYQQEASKLR AQISNLQNQNRNMLGESLGALSLRELKNLESRVERGIS RIRSKKNELLFAEIEYMQKRQEIDLHHNNQYLRAKIAE SERVQGQHMNLMPGGSSGYEQLVETQPFDARNYLQV NGLQPNNDYPRQDQLPLQLV
AmPLE	S53900	*Antirrhinum majus*	MEFPNQDSESLRKNGRGKIEIKRIENITNRQVTFCKRR NGLLKKAYELSVLCDAEVALVVFSSRGRLYEYANNSV RATIERYKKASADSSNSVSTSEANTQFYQQEANKLRRQ IREIQTSNRQMLGEGVSNMALKDLKSTEAKVEKAISRI RSKKNELLFAEIEHMQKRELELHNANMFLRAKIAEGE

			RAQQQMNLMPGSDYQPMTSQSYDVRNFLPMNLMEP NQQQYSRHDQTALQLV
AtAG	X53579	*Arabidopsis thaliana*	TAYQSELGGD SSPLRKSGRG KIEIKRIENT TNRQVTFCKR RNGLLKKAYELSVLCDAEVA LIVFSSRGRL YEYSNNSVKG TIERYKKAIS DNSNTGSVAEINAQYYQQES AKLRQQIISI QNSNRQLMGE TIGSMSPKEL RNLEGRLERSITRIRSKKNE LLFSEIDYMQ KREVDLHNDN QILRAKIAEN ERNNPSISLM PGGSNYEQLM PPPQTQSQPF DSRNYFQVAA LQPNNHHYSS AGRQDQTALQLV
AtSHP1	M55550	*Arabidopsis thaliana*	MEEGGSSHDA ESSKKLGRGK IEIKRIENTT NRQVTFCKRR NGLLKKAYELSVLCDAEVAL VIFSTRGRLY EYANNSFIYL LLEKKKKKKK KKNLWIYSSHVVRGTIERYK KACSDAVNPP SVTEANTQYY QQEASKLRRQ IRDIQNSNRHIVGESLGSLN FKELKNLEGR LEKGISRVRS KKNELLVAEI EYMQKREMEL QHNNMYLRAK IAEGARLNPD QQESSVIQGT TVYESGVSSH DQSQHYNRNYIPVNLLEPNQ QFSGQDQPPL QLV
AtSHP2	M55553	*Arabidopsis thaliana*	MEGGASNEVA ESSKKIGRGK IEIKRIENTT NRQVTFCKRR NGLLKKAYELSVLCDAEVAL VIFSTRGRLY EYANNSVRGT IERYKKACSD AVNPPTITEANTQYYQQEAS KLRRQIRDIQ NLNRHILGES LGSLNFKELK NLESRLEKGISRVRSKKHEM LVAEIEYMQK RVKEIELQND NMYLRSKITE RTGLQQQESS
NbAG	JQ699177	*Nicotiana tabacum*	MEFQSDLTREISPQRKLGRGKIEIKRIENTTNRQVTFCK RRNGLLKKAYELSVLCDAEVALIVFSSRGRLYEYANNS VKETIERYKKACSDSSNTDSISEANAQYYQQEASKLRA QIGNLQNKNRNMLGECLAALSLRDLKNLEQNIEKGIS KIRSKKNELLFAEIEYMQKREIDLHNNNQYLRAKIAET ERAQQQQQQMNLMPGSSSYELVHPPQQFDTRNYLQ VNGLQTNDHYTRQDQPSLQLV
NbSHP	JQ699178	*Nicotiana tabacum*	MEFPNEEFESSNSQRKSGRGKIEIKRIENTTNRQVTFCK RRNGLLKKAYELSVLCDAEVALIVFSSRGRL YEYANNSVRATIDRYKKHHADSTSQGSVSEANTQYYQ QEAAKLRRQIRDIQTYNRQIVGEALSSLSPRDL KNLEGKLEKAIGRVRSKKNELLFSEIEVMQKREIEMQN ANMYLRAKIAEVERAQQQMNLMPGGSEYSHHQ QQPMSTSQNYNDARNFLPVNLLEPNPHYSRHDDQTA LQLV
TAG1	L26295	*Lycopersicon esculentum*	MDFQSDLTREISPQRKLGRGKIEIKRIENTTNRQVTFCK RRNGLLKKAYELSVLCDAEVALVVFSNRGRLYEYANN SVKATIERYKKACSDSSNTGSVSEANAQYYQQEASKLR AQIGNLMNQNRNMMGEALAGMKLKELKNLEQRIEK GISKIRSKKNELLFAEIEYMQKREVDLHNNNQYLRAKI AETERAQHQHQQMNLMPGSSSNYHELVPPPQQFDTR NYLQVNGLQTNNHYPRQDQPPIQLV
TAGL1	AY098735	*Lycopersicon esculentum*	MVFPINQELLVDESSSQLRKTSGGTGGGGRGKIEIKRIE NTTNRQVTFCKRRNGLLKKAYELSVLCDAEVSLIVFSS RGRLYEYANNSVRATIDRYKKHHADSTSTGSVSEANT QYYQQEASKLRRQIRDIQTYNRQIVGEALGSLSPRDLK NLEGKLEKAIGRVRSKKNELLFSEIELMQKREIELQNA NMYLRAKIAEVERAQEQMNLMPGGGGGGGGGGG GSDHQYHHQPNYEDARNNSLPVNLLEPNPHYSRRDN GDQTPLQLV

PMADS3	X72912	*Petunia hybrid*	MEFQSDLTREISPQRKLGRGKIEIKRIENTTNRQVTFCKRRNGLLKKAYELSVLCDAEVALIVFSSRGRLYEYANNSVKATIERYKKACSDSSNTGSIAEANAQYYQQEASKLRAQIGNLQNQNRNFLGESLAALNLRDLRNLEQKIEKGISKIRAKKNELLFAEIEYMQKREIDLHNNNQYLRAKIAETERSQQMNLMPGSSSYDLVPPQQSFDARNYLQVNGLQTNNHYPRQDQPPLQLV
FBP6	X68675	*Petunia hybrid*	MVFPNQEFESSSSQRKSGRGKIEIKRIENTTNRQVTFCKRRNGLLKKAYELSVLCDAEVALIVFSSRGRLYEYANNSVRATIDRYKKHHADSTSTGSVSEANTQYYQQEAAKLRRQIRDIQTYNRQIVGEALSSLSPRGLKNLEGKLEKAIGRVRSKKNELLFSEIELMQKREIEMQNANMYLRAKIAEVERATQQMNLMHGGGSEYQQQPMSSTSQPYDARNFLPVNLLEPNPHYSRQDQTALQLV
DcaAGa		*Dianthus caryophyllus*	MEFSSQITREEGSPSSQRKLGRGKIEIKRIENTTNRQVTFCKRRNGLLKKAYELSVLCDAEVALIVFSSRGRLYEYANHSVKGTIERYKKACSDSTGAGSVAEANAQYYQQEAAKLRGQIRTITDSNRLLSRQLMGEGLSDLSMKELKNLESKLEKGISRIRSKKNELLFAEIEFMQKREIDLHNHNQFLRAKIAENERAQQSMRLMPGGSSEYELAPPPQSFDSRNYFQVNALQPNEHYSRQDQTPLQLV
DcaAGb		*Dianthus caryophyllus*	MEFSSQITREEGSPSSQRKLGRGKIEIKRIENTTNRQVTFCKRRNGLLKKAYELSVLCDAEVALIVFSTRGRLYEYANHSVKGTIERYKKTCSDTSTGSVAEANAQYYQQESAKLRSQIRTMTESNRSLSRHMMGEGLTGLNMKELKNLETKLEKGISRIRSKKNELLFAEIEFMQKREVDLHNNNQLLRAKIAENERAQQSMSLMPGGGDYELAPPPQSFDSRNYFQVNALQPNEQYSCQDQTPLQLV
Bv8_188310_mhzu.t1		*Beta vulgaris*	MEGSPSSQRKMGRGKIEIKRIENTTNRQVTFCKRRNGLLKKAYELSVLCDAEVALIVFSSRGRLYEYANHSVKGTIDRYKKACSDQSGAGSVAEANAQYYQQEAAKLRNQIRTATENNRLLSRHMMGEGLSSLSMKELKNLETKLERGISRIRSKKNELLFAEIEFMQKREIELHNNNQFLRARISENERAQQSMSLMPGGSDYDLVPSQSFDSRNYFQVNALQPSSQYARQDQTPLQLV
Spo09086		*Spinacia oleracea*	MLTKDVKGTIDRYKKACSDQTGAGSVAEANAQYYQQEAAKLRNQIRTATENNRTNYLKWNNRLLSRHMMGEGLSSLSMKELKNLETKLEKGISRIRSKKNELLFAEIEFMQKREIELHNNNQFLRARISENERAQQSMSLMPPGGSDYDLVPSQSFDSRNYFQVNALQPNTTVSNLSTCAWRRKHKPSFATGDQVVAGAWCLVPGAWCLVPGAWWWFLNCHFAVVDVT
AUR62035850-RA		*Chenopodium quinoa*	MEFPTQVMEEGSPSSQRKMGRGKIEIKRIENTTNRQVTFCKRRNGLLKKAYELSVLCDAEVALIVFSSRGRLYEYANHSVKGTIDRYKKACSDQTGAGSVAEANAQNGKINVTEFPRPRNVIWYYQQEAAKLRQSIKSQTNTNRHMMGEGLSSLSMKELKNLESKLEKGINRIRSKKNELLFAEIEFMQKREIELHNNNQFLRARISENERAQQSMSLMPGGSDYDLVPSQSFDSRNYFQVNALQPNNQYARQDQTPLQLV
AUR62027653-RA		*Chenopodium quinoa*	MEFPTQVMEEGSPSSQRKMGRGKIEIKRIENTTNRQVTFCKRRNGLLKKAYELSVLCDAEVALIVFSSRGRLYEYANHSVKGTIDRYKKACSDQTGAGSVAEANAQYYQQEAAKLRGQIKTATENNRHMMGEGLSSLSMKELKNLETKLEKGINRIRSKKNELLFAEIEFMQKREIELHNNNQFLRARISENERAQQSMSLMPGGSDYDLVPSQSFDSRNYFQIELAGAESDQVNSLMNGI

VvAG	NM_001281168	*Vitis vinifera*	MGRGKIEIKRIENTTNRQVTFCKRRNGLLKKAYELSVL CDAEVALIVFSSRGRLYEYANNSVKSTIERYKKASADSS NTGSVSEANAQFYQQESSKLHQQIRNLQNSNRHMLG ESLGSLNFKDLKSLEIRLEKGISRIRSKKNELLFAEIEYM QKREIDLHNDNQYLRARIAENERNEQQMSLMPGGAN YELMPSQQFDSRNYFQLNGLQPNQSYSRQDQPALQLV
VvMADS1	NP_001268105	*Vitis vinifera*	MGRGKIEIKRIENTTNRQVTFCKRRNGLLKKAYELSVL CDAEVALIVFSSRGRLYEYANNSVRTTIERYKKVCSDSS NTGSVSEANAQFYQQEASKLRRQIRDIQNLNRHILGE ALSSLNFKELKNLETRLEKGISRIRSKKNELLFAEIEYM QKREIELQNSNLFLRAQIAENERAQQQMNLMPGSQY ESVPQQPYDSQNLLPVNLLDPNHHYSRHDQTALQLV
KjAG	AMQ23646	*Kerria japonica*	MAYENKSMSMAIDSPQRKLGRGKIEIKRIENTTNRQVT FCKRRNGLLKKAYELSVLCDAEVALIVFSNRGRLYEYA NNSVRATVERYKKACADTSNNGSVSEASTQYYQQEA AKLRAQIGNLQNSSRHMLGESLSSMTMKDLKSLEGKL EKGISRIRSKKNELLFTEIEYMQKRELDLHNNNQLLRA KIAENERSQQNINVMAAGGGNYEIMQSQPYDSRNYF QVSALQPNHQYNPRQDQIALQLV
AtSTK	NP_001329612	*Arabidopsis thaliana*	MMGRGKIEIKRIENSTNRQVTFCKRRNGLLKKAYELSV LCDAEVALIVFSTRGRLYEYANNNIRSTIERYKKACSDS TNTSTVQEINAAYYQQESAKLRQQIQTIQNSNRNLMG DSLSSLSVKELKQVENRLEKAISRIRSKKHELLLVEIENA QKREIELDNENIYLRTKVAEVERYQQHHHQMVSGSEI NAIEALASRNYFAHSIMTAGSGSGNGGSYSDPDKKILH LG
FBP11	CAA57445	*Petunia hybrid*	MGRGKIEIKRIENNTNRQVTFCKRRNGLLKKAYELSVL CDAEIALIVFSTRGRVYEYANNNIKGTIERYKKATAETS NACTTQELNAQFYQQESKKLRQQIQLLQNTNRHLVG EGLSALNVRELKQLENRLERGITRIRSKKHEMILAETE NLQKREIQLEQENTFLRSKIAENERLQELSMMPATGQE YNAFQQYFARNMLQLNMMEGGVPSYDPLPAHDKKS LQLE
CUM1	AAC08528	*Cucumis sativus*	MSKHYQSPLTRMIKEEGKGKLQIKGMFQNQEEKMSDS PQRKMGRGKIEIKRIENTTNRQVTFCKRRNGLLKKAY ELSVLCDAEVALIVFSSRGRLYEYANNSVKATIDRYKK ASSDSSNTGSTSEANTQFYQQEAAKLRVQIGNLQNSN RNMLGESLSSLTAKDLKGLETKLEKGISRIRSKKNELLF AEIEYMRKREIDLHNNNQMLRAKIAESERNVNMMGG EFELMQSHPYDPRDFFQVNGLQHNHQYPRQDNMAL QLV
CUM10	AAC08529	*Cucumis sativus*	MGRGKIEIKRIENTTNRQVTFCKRRNGLLKKAYELSVL CDAEVALIVFSSRGRLYEYSNNSIKTTIERYKKACSDSS ATSSVTELNTQYYQQESAKLRQQIQMLQNSNSNLVRH LMGDSLSALTVKELKQLENRLERGITRIRSKKHEMLLA EIEYLQKREIELENENVCIRTKIAEVERVQQANMVSGQ ELNAIQALANSRNFFSPNIMEPAGPVSYSHQDKKMLH LG
GhMADS3	XP_016711294	*Gossypium hirsutum*	MVYPNESLEDSPQKKMGRGKIEIKRIENTTNRQVTFCK RRNGLLKKAYELSVLCDAEVALVVFSSRGRLYEYANN SVKATIERYKKASDSSNTGSVAEVNAQFYQQEADKLR NQIRNLQNANRHMLGESIGGLPMKELKSLESRLEKGIS RIRSKKNELLFAEIEYMQKREIDLHNNNQLLRAKIAEN ERKQQSMNLMPGGSSANFEALHSQPYDSRNYFQVDA LQPATNYYNPQLQQDQIALQLV
GhMADS5	ABM69043	*Gossypium hirsutum*	MGRGKIEIKRIENTTNRQVTFCKRRNGLLKKAYELSVL CDAEVALIVFSTRGRLYEYSNNNIRSTIERYKKACSGTS NTNTVTEINAQYYQQESAKLRQQIQMLQNSSRHLMG

			DSLSSLTVKELKQLENRLERGITRIRSKKHEMLLAEIEYFQKREVELENESVCLRAKIAEIERVEEANMVTGAELNAIQALASRNFFTPNVIERGTPTPYSHHDKKILHLG
GhMADS7	ABM69045	*Gossypium hirsutum*	MEFPNLDPESSSQKKMGRGKIEIKRIENTTNRQVTFCKRRNGLLKKAYELSVLCDAEVALIVFSSRGRLYEYANNSVRATIERYKKACSDATTPGSVAEANIQFYQQEATKLRRQIRDVQNMNRHILGEALSSLTFKELKNLEGRLEKGICRIRSKKNELLFAEIGFMQKREVELQNDNMYLRAKIAENERAQQQSNQLMQAASSYNRNFLPVNLLEPSNNDYSNQDQTPLQLV
GsAG1	LC022775	*Gentiana scabra*	MDYPPRQEFDSSSSRKSGRGKIEIKRIENATNQQVTFCKRRSGILKKAYELSVLCDAEVALIVFSTRGRLYEYASSSVRGTIERYKKACADTTNSGSISEANTQFYQQQSNKLRKDIKEIQKANRNMLGEGVESIQPKDLKKIEGNLERAIGKIRTRKNELLFAEIELMQKREMELQNANLYLRAKNFDMVQIAENERATTDPHMNLMPASASEYNHHQSMASHSFDDVRSFIPVNLLEPNQHYSRQDPTALQLV
GsAG2	LC022779	*Gentiana scabra*	MDSQNQEIESSNSRKSGRGKIEIKRIENKTNRQVTFCKRRSGLLKKAYELSVLCDAEVALIVFSSSGRLYEYANNSAKGTIERYKKACGDSTSAGSVSEANIQFYQQEANQLRKNIRDIQSSNRHILGEGLDELSFKQIKNLEGRVEKGIARVRSRKNELLAAEIELMKKREIELQNANLYLRAKITENDQQRVQAEQQQMNFMPASDYQTNNNNIASEPNYQEVHNFIPVNFLDHNQHYSSQDPTALQFV
GsSTK1	LC022768	*Gentiana scabra*	MGRGKIEIKRIENNTNRQVTFCKRRNGLLKKAYELSVLCDAEVALVVFSSRGRVYEYANNNIRATIDRYRKATSDVPTVFTTQEINAQFYQQESKKLRQQIQILQNSNRHLMGEGLSALNVKELKQLETRLERGLSRIRSKKHEMILAEAEYLQKREIQLEQENAWLKAKISEQEKLQHLSMMPPGQEYNEAMEAYFAQNMLQLNMMEGVPIYSLSSDKKSLHLG

Table S3: The sequences of primers used for semi-quantitative PCR and qRT-PCR analysis in carnation.

Gene name	Efficiency	Oligo Name	Primer sequence（5′–3′）
DcaAGa	98.9%	*DcaAGa*-F	CGAATCCGATCCAAAAAGAATG
		DcaAGa-R	TCGTACTCGCTGCTACCTCC
DcaAGb	97.8%	*DcaAGb*-F	GCATTAGTAGAATCCGATCCAA
		DcaAGb-R	TCGTAGTCACCACCACCCG
DcaAP1	99.1%	*DcaAP1*-F	ACAAGGTGTTGGTGATGA
		DcaAP1-R	GTATTGGCGATTTGGCTC
DcaAP2	98.3%	*DcaAP2*-F	AGCCAAACACACTTACAATCAC
		DcaAP2-R	TCCCCCGTTGCTGCTAC
DcaSEP1	99.6%	*DcaSEP1*-F	GGTGAAAGATAGTCAAATGAGGCAG
		DcaSEP1-R	GTCAATGGGTTGGAAAAGAGGTT
DcaSEP3	98.6%	*DcaSEP3*-F	ATCTTCGCAGTGGAACGGACAA
		DcaSEP3-R	CCTGCTGATAACCGATTTGTAAG
DcaPI	97.9%	*DcaPI*-F	GGAGGAGAATAATCAGCTTGTA
		DcaPI-R	GATGGAATCGGGTGCTGA
DcaAP3	99.6%	*DcaAP3*-F	CTACAAAGAAAAAGGTGCGAAATGG
		DcaAP3-R	GCAATCTGAGAGCGAGCAAACGAG
DcaGAPDH	98.6%	*DcaGAPDH*-F	CGGAAAGTTGACTGGTATGGC
		DcaGAPDH-R	CATCCTCGGTGTAGCCCAAAAT
Dcaβ-actin	97.6%	*Dcaβ-actin*-F	AAACTTTCAACGCTCCTGCTATG
		Dcaβ-actin-R	CTATCCCTTACTATTTCTCGCTCA

Table S4: The sequences of primers used for semi-quantitative PCR and qRT-PCR in transgenic *Arabidopsis*.

Gene Name	Oligo Name	Primer Sequence（5′–3′）
AtPI	*AtPI* -F	AAGAGGATAGAGAACGCAAACA
	AtPI -R	GAAGGACAACAGTAATCAATCA
AtAP3	*AtAP3*-F	AGAGAACCAGACAAACAGACAAG
	AtAP3-R	CTTCGTTGTGGTGTTAGGGCTGA
AtAG	*AtAG* -F	CTACGAGCAGCTTATGCCACCA
	AtAG -R	GAGTAATGGTGATTGTTAGGTTGC
AtSTK	*AtSTK* -F	TAGGATGGGAAGAGGAAAGATAGAA
	AtSTK -R	TCTTATGTTGTTATTGGCGTATTCA
AtSEP1	*AtSEP1*-F	CCTTATCTCAAGTGTCTGCG
	AtSEP1-R	GAGAGGAGAACCAGAAGCGA
AtSEP2	*AtSEP2*-F	GATTTAGCCGAGAACGGGAAGAT
	AtSEP2-R	ACTCTCTATGTAATCCGTAAGCG
AtSEP3	*AtSEP3*-F	GTATCAGGGGCAACAAGATGGAAT
	AtSEP3-R	AAAGAGAGGGATTGATTAAGTGAGAA
AtACT2	*AtACT2*-F	GGCTCCTCTTAACCCAAAGGC
	AtACT2-R	CACACCATCACCAGAATCCAG
AtACT11	*AtACT11*-F	CCACATGCTATTCTGCGTTTGGACC
	AtACT11-R	CATCCCTTACGATTTCACGCTCTGC
